# Equity Over Equality: Post-Brexit Demographic Change, Workforce Pressures, and the Future of Equitable Primary Care in England

**DOI:** 10.7759/cureus.117923

**Published:** 2026-10-14

**Authors:** Trimanjot Singh Anand, Sakshi Watarkar, Vishesh Singh

**Affiliations:** 1 Internal Medicine, University Hospitals of Leicester NHS Trust, Leicester, GBR; 2 School of Medical Sciences, University of Leicester, Leicester, GBR; 3 General Surgery, University Hospitals of Leicester NHS Trust, Leicester, GBR

**Keywords:** brexit, cultural competency, digital health, general practice, health-care equity, migration, multimorbidity, nhs workforce, primary care, proportionate universalism

## Abstract

General practice (GP) in England faces structural pressures that incremental reform is unlikely to resolve: a contracting GP workforce, a rising burden of multimorbidity, digital transformation that risks widening access inequalities, and a post-Brexit change in the composition of migration. This narrative review, reported with reference to the Scale for the Assessment of Narrative Review Articles (SANRA), asks whether the current model of general practice in England is configured to deliver equitable care over the coming three decades and what structural changes the evidence supports. Published and grey literature from MEDLINE, Embase, the Cochrane Library, Google Scholar, and national policy sources was synthesised thematically. Three cross-cutting themes are examined. First, the post-Brexit shift in migration: by 2021, 16.8% of residents of England and Wales were born outside the UK, and since the end of European Union (EU) free movement, net migration has been driven by non-EU nationals while EU net migration has turned negative, with implications for culturally and linguistically competent care. Second, the distinction between equality and equity, framed by Marmot's principle of proportionate universalism. Third, the transformation required of the GP role within integrated neighbourhood teams. Observational evidence consistently associates personal continuity of care with lower mortality, and cross-national comparisons associate stronger primary care with better population health; workforce and access inequalities along deprivation lines have widened. Prospective evidence that specific reform models work at NHS scale remains limited. We conclude that three interdependent changes are indicated: integration into multidisciplinary neighbourhood teams that protect continuity, contractual reform that weights resources for deprivation, multimorbidity, and language need, and a workforce strategy built as much on retention and cultural safety as on recruitment. We propose measurable indicators for each. The trained generalist, able to hold undifferentiated clinical complexity over time, is likely to remain central to whatever model emerges.

## Introduction and background

General practice in England delivers more patient contacts than any other part of the NHS, yet receives a comparatively small share of NHS spending: 8.3% in 2024-2025, down from 8.8% in 2015-2016 [1]. NHS workforce statistics show that the number of fully qualified full-time-equivalent (FTE) GPs in England fell after 2015, while the registered population grew older and the burden of long-term conditions increased [2]. As early as 2016, the King's Fund reported that general practice workload had outgrown the funding and workforce available to meet it [3]. The intervening decade has produced much policy commentary but limited structural change.

The problem is not only one of funding or staffing numbers, though both are inadequate. Most practices in England hold the General Medical Services (GMS) contract, under which core funding follows weighted capitation: a fixed annual payment per registered patient, adjusted for factors such as age and deprivation. This is supplemented by the Quality and Outcomes Framework (QOF), a pay-for-performance scheme based largely on disease-specific indicators. The basic unit of clinical exchange remains the short face-to-face appointment, typically around ten minutes. Since 2019, practices have also been grouped into primary care networks (PCNs) of roughly 30,000-50,000 patients, funded through the Additional Roles Reimbursement Scheme (ARRS) to employ pharmacists, physiotherapists, social prescribers, and other staff. This model was designed for a population with shorter life expectancy and less multimorbidity. In our view, the mismatch between the model and current epidemiology is likely to contribute to avoidable harm, although direct causal evidence for this is limited.

The COVID-19 pandemic exposed these fault lines. The shift to remote and digital consultation showed that general practice could adapt quickly, but not evenly. A systematic review of remote consultations found that internet-based consultations were used more by younger, more affluent, and more educated patients, and that remote modes were used less by immigrants [4]. NHS England estimates that around ten million adults in England lack foundation-level digital skills [5]. Without compensating measures, a digital-first model risks allocating access by digital capability rather than clinical need.

Brexit added a layer of complexity that the primary care literature has been slow to engage with. In the 2021 Census, 10.0 million residents of England and Wales (16.8%) were born outside the UK, up from 13.4% in 2011 [6]. Since European Union (EU) free movement ended in January 2021, non-EU nationals have driven net migration. Total net migration has since fallen sharply, to a provisional 171,000 in the year ending December 2025, but non-EU net migration remained positive at 350,000 while EU and British net migration were negative [7]. Migration Observatory analysis describes this as a lasting change in composition rather than only in volume [8]. For general practice, the composition matters most: it changes the languages, health beliefs, and prior health-system experience of the populations presenting, particularly in urban England.

Scope

This review focuses on England. Health is devolved within the UK, and general practice contracts, funding, and workforce policy differ between England, Scotland, Wales, and Northern Ireland. Where England-specific data were unavailable, we used UK-wide migration statistics, England-and-Wales Census data, or, for multimorbidity epidemiology, a large Scottish study; we flag these instances in the text. International evidence is used to inform, not replace, English findings.

Throughout, we distinguish equality, meaning the same provision for every patient, from equity, meaning provision proportionate to need so that patients can achieve comparable outcomes. This narrative review asks: is the current model of general practice in England configured to deliver equitable care over the next three decades, and what structural changes does the evidence support? Three themes run through the analysis: the epidemiological challenge of multimorbidity in an ageing population; the demographic and cultural implications of post-Brexit population change; and the shift from equality to equity as the organising logic of service design. We do not argue for dismantling general practice, but for evidence-informed transformation, with the trained generalist at its centre and equity as its measure of success.

Methods

Design and Reporting

This is a narrative review, reported with reference to the Scale for the Assessment of Narrative Review Articles (SANRA) [9]. A systematic review following PRISMA (Preferred Reporting Items for Systematic Reviews and Meta-Analyses) guidelines was not undertaken because the relevant evidence spans epidemiological studies, trials, workforce statistics, migration data, and policy documents, which are too heterogeneous in design and purpose for systematic synthesis.

Sources and Dates

We searched MEDLINE, Embase, the Cochrane Library, and Google Scholar between January and May 2025, with an updated search during revision (to October 2026) for material published in 2025-2026. Grey literature was searched on the websites of NHS England, NHS Digital, the Department of Health and Social Care, the King's Fund, the Health Foundation, the Institute of Health Equity, the Migration Observatory at the University of Oxford, the Office for National Statistics (ONS), the British Medical Association, the General Medical Council, and Doctors of the World UK. Reference lists of key papers were hand-searched.

Search Strategy

Search terms were organised into three clusters (Table 1) and combined with the Boolean operator AND across clusters where appropriate. Foundational literature had no date restriction; priority was given to studies from 2010 onwards, with targeted searches for 2020-2026 material on post-Brexit migration, artificial intelligence, digital inclusion, and GP workforce retention. 

**Table 1:** Search term clusters

Cluster	Example terms (combined with OR within each cluster)
1. Primary care organisation and delivery	"general practice", "primary care", "family medicine", "general practitioner*", multimorbid*, "continuity of care", workforce, burnout, retention, "primary care network*", capitation, "Quality and Outcomes Framework"
2. Demographic and equity considerations	equity, inequalit*, "inverse care law", "proportionate universalism", deprivation, migra*, Brexit, asylum, interpret*, "cultural competen*", "digital exclusion", "digital inclusion"
3. Future models of care	"integrated care", "neighbourhood team*", "artificial intelligence", "remote consultation*", "video consultation*", telemedicine, "health system performance"

Eligibility

We included English-language peer-reviewed studies (systematic reviews, trials, cohort and cross-sectional studies, and qualitative studies), national health policy documents, official statistics, and reports from established health policy institutes and professional bodies, where they addressed general practice or primary care in England, the UK, or comparable high-income health systems. We excluded opinion pieces without an evidence base, studies confined to secondary care, and sources from health systems without a registered-list primary care model unless they addressed a cross-national question directly.

Selection

Titles and abstracts were screened, and full texts of potentially relevant sources were read by the authors; uncertain items were resolved by discussion. As is common for narrative reviews, a formal screening log was not kept, so numbers of records screened are not reported. The final review cites 50 sources.

Appraisal

No formal risk-of-bias tool was applied because the included sources range from randomised trials to policy documents and no single tool covers them all. Instead, for each domain, we graded the strength of evidence by design (systematic reviews and trials highest; large cohort and cross-sectional studies; surveys and qualitative studies; policy analyses and expert opinion lowest), summarised in Table 2. Causal language is reserved for trial evidence.

Synthesis

Key findings from each source were extracted into a matrix by domain. Through iterative reading, the authors grouped findings into six domains (multimorbidity, workforce, technology, migration, equity and funding, and neighbourhood models), which were refined by discussion into three cross-cutting themes. Empirical findings are distinguished in the text from the authors' interpretation and recommendations.

## Review

The epidemiological transition: multi-morbidity and the structural limits of the episodic model

Barnett and colleagues' cross-sectional study of 1.75 million patients registered with 314 Scottish practices found that 23.2% had two or more long-term conditions; onset occurred 10-15 years earlier in the most deprived areas, and combined physical and mental health conditions were substantially more common in deprived populations [10]. We cite this Scottish study because no single-source English study of comparable size exists; differences between the health systems limit direct transfer, but the age and deprivation gradients it describes are likely to apply in England. Multimorbidity is not simply more conditions to manage. It requires coordination across conditions, which single-disease guidelines and short single-problem appointments were not designed to provide [11]. In our interpretation, when one short appointment must address, for example, type 2 diabetes alongside depression, chronic obstructive pulmonary disease, and osteoarthritis, competing demands make it likely that the mental health component is deprioritised and that medications are optimised condition by condition, adding to treatment burden [11].

The intervention evidence requires careful interpretation. Salisbury and colleagues' cluster-randomised trial of the patient-centred 3D approach (1,546 patients in 33 practices in England and Scotland) found no difference in the primary outcome, health-related quality of life, but did improve patients' experience of patient-centred care [12]. Observational evidence suggests that continuity, a core component of such models, is associated with fewer emergency admissions for ambulatory care-sensitive conditions [13]. Together, these findings suggest that whole-person care may yield benefits in experience, coordination, and admissions sooner than in quality-of-life or biomarker outcomes. Evaluation frameworks that rely narrowly on biomarker targets therefore risk undervaluing such care. More appropriate indicators would include continuity indices, patient-reported experience and treatment burden, completion of an annual whole-person review with a single care plan, and ambulatory care-sensitive admissions.

Roland and Paddison identified QOF as one structural driver of fragmented multimorbidity care: by rewarding discrete disease targets rather than holistic outcomes, it steers clinical attention toward single-condition metrics [11]. The clinical competencies needed for complexity, namely relational continuity, longitudinal knowledge, and coordinated oversight, already exist within general practice. The problem, as we see it, is a delivery architecture of short appointments and fragmented lists that limits how deeply those competencies can be used, and a payment system that does not reward them.

Workforce sustainability: the unravelling of the partnership model and the evidence on retention

BMA data show a sustained decline in the number of GPs choosing partnership, with growing numbers in salaried or locum roles [14]. The partnership model asks clinicians to accept personal financial liability for premises and staff alongside demanding clinical work. Karuna and colleagues' meta-analysis of 60 studies (22,177 GPs, 29 countries) reported burnout prevalence ranging from 6% to 33% across burnout dimensions, with substantial heterogeneity that suggested country-specific factors matter [15]. A separate systematic review of determinants found that factors specific to the nature and organisation of GP work contributed more to burnout than generic factors [16]. Bodenheimer and Sinsky's quadruple aim frames clinician wellbeing as a performance variable [17], and a systematic review found that poor staff wellbeing and burnout are associated with poorer patient safety [18].

The evidence on what retains GPs is less developed than the evidence on why they leave. A systematic review of qualitative studies found that GPs' decisions to leave direct patient care were driven by falling job satisfaction, linked to changing doctor-patient relationships and loss of autonomy, combined with rising workload and feeling unsupported [19]. We infer from this that protected non-clinical time, team-based working, reduced administrative burden, and clear career pathways are plausible retention levers, although robust trial evidence is lacking. Workforce distribution is also unequal: by December 2020, practices in the most deprived areas of England had 1.4 fewer FTE GPs per 10,000 patients than those in the least deprived, and the gap had widened since 2015, while deprived areas had more pharmacists and physician associates [20]. Recruitment alone is unlikely to close this gap while working conditions in high-deprivation practices remain more demanding.

Technology as enabler and risk: AI, digital consultation, and equity

The 2019 Topol Review argued that AI, genomics, and digital tools would reshape clinical workflows within a decade [21]. A 2026 Primary Care review describes AI already used in documentation, triage, and risk stratification, and highlights gaps in evaluation, governance, and equity safeguards [22]. Adoption is outpacing evidence: in a February 2024 survey of 1,006 UK GPs, 20% reported using generative AI in clinical practice, despite little formal guidance [23].

On remote consultation, the evidence points in a consistent direction. The VOCAL (Virtual Online Consultations: Advantages and Limitations) study found video consultations safe and acceptable for selected patients and conditions, but dependent on technical and organisational infrastructure [24]. Parker and colleagues' systematic review found unequal use of remote modes by age, affluence, education, and immigrant status, and no studies reporting effects on quality or clinical outcomes [4]. NHS England links lack of digital skills to a higher risk of health inequality [5]. The overlap between the digitally excluded and the clinically most vulnerable reflects the social gradient that Hart described in 1971 as the inverse care law [25]. A digital strategy without compensating mechanisms is therefore not neutral; in effect, it rations care by digital capability.

Algorithmic bias deserves specific attention. Dermatology AI models have shown lower diagnostic performance on darker skin tones [26], and a widely used US population-health algorithm underestimated the needs of Black patients because it used healthcare cost as a proxy for need [27]. Cardiovascular risk scores derived in largely White populations have misestimated risk in some minority ethnic groups, which is why QRISK3 incorporates ethnicity [28]. Deploying AI in diverse, high-deprivation settings without equity auditing risks systematising such errors. Existing tools could be used for this: the Health Equity Assessment Tool (HEAT) [29] and NHS England's digital inclusion framework [5]. We propose that any AI or digital access tool in general practice report performance and uptake by ethnicity, deprivation, age, and preferred language before and after deployment.

Post-Brexit demographic change and the cultural competency imperative

Before the 2016 referendum, EU nationals made up a large share of UK net migration. Since free movement ended in 2021, EU net migration has been negative and non-EU nationals have driven the totals [7,8]. Total net migration has fallen since its 2022-2023 peak, so the volume is volatile; the composition, however, has shifted durably, and 16.8% of the population of England and Wales was born abroad by 2021 [6]. For general practice, this means more patients whose first language is not English, who are unfamiliar with the NHS, or whose entitlement is uncertain.

Rechel and colleagues' review documented consistent patterns across Europe: poorer primary care access, more undetected chronic disease, lower uptake of preventive services, and underuse of mental health support among migrants, driven by language barriers, unfamiliarity with health systems, uncertain entitlement, and differing illness frameworks [30]. The UK evidence base has significant gaps: most studies rely on self-reported access, and prospective UK data linking language or cultural barriers to clinical outcomes are sparse. The case for culturally competent primary care in England therefore rests on European epidemiological evidence and plausible mechanisms rather than large UK outcome studies.

Betancourt and colleagues' framework defines cultural competence as intervention at organisational, structural, and clinical levels [31]. In our assessment, English general practice falls short at each level. Interpreter provision is commissioned locally and inconsistently, despite national guidance [32]. Telephone and video consultation have expanded without equivalent provision for patients needing face-to-face interpretation. Training in culturally responsive consultation remains variable. Registration barriers also persist: NHS England guidance states that proof of address, identity, or immigration status is not required to register with a GP [33], yet Doctors of the World found that 19% of 2,189 attempts to register patients in 2018 were wrongly refused [34]. Equity indicators such as access by preferred language and interpreter use are not routinely measured nationally, and we propose that they should be.

Brexit also affects the workforce. Future recruits are increasingly likely to come from outside the EU, from different training and regulatory systems. The GMC's "Fair to Refer?" report found that international medical graduates are referred by employers for fitness-to-practise concerns at disproportionate rates, partly because of inadequate induction and support [35]. A workforce strategy that does not design in cultural safety and equitable progression for these doctors is unlikely to retain them.

Equity versus equality: proportionate universalism and the gap between policy and practice

In a population where disease burden, digital access, language fluency, and financial resilience are distributed unevenly, equal provision does not produce fair outcomes; it reproduces the disadvantage Hart described [25]. Marmot's principle of proportionate universalism, set out in 2010 and reaffirmed in the 2020 ten-year review, offers the most developed framework for operationalising equity: universal services delivered with intensity proportional to disadvantage [36,37].

The NHS Core20PLUS5 framework, launched in 2021, is the most specific current attempt to apply this principle, targeting the most deprived 20% of the population and locally defined groups across five clinical priority areas [38]. Its limitation is that it is a targeting framework, not a resourcing mechanism: it identifies where support should be intensified but does not change the funding formula to provide it. Without that, it risks becoming a way of monitoring a gap that policy is not resourced to close.

Starfield, Shi, and Macinko's review concluded that stronger primary care is associated with better population health and lower costs, with the greatest benefits in disadvantaged populations [39]; Macinko and colleagues' analysis of OECD (Organization for Economic Cooperation and Development) countries between 1970 and 1998 reached consistent conclusions [40]. These findings are observational but have been available for over two decades. In England, the Health Foundation found that practices serving more deprived populations receive around 7% less funding and have fewer GPs per need-adjusted patient than those in less deprived areas, and called for a review of the Carr-Hill allocation formula [41], consistent with the workforce gap described above [20]. Reweighting GMS capitation explicitly for deprivation, multimorbidity, and language need is evidentially justified, though it requires a political commitment to redistribution that has proved easier to state than to implement.

Towards an integrated neighbourhood model: evidence, limitations, and the generalist’s irreplaceable role

The 2022 Fuller Stocktake set out integrated neighbourhood teams: multidisciplinary teams from general practice, community, mental health, and social care serving populations of around 30,000-50,000, with a shift from reactive appointments to proactive, relationship-based care [42]. This model built on PCNs introduced by the NHS Long Term Plan [43], and the 2025 10 Year Health Plan for England makes a neighbourhood health service central to national policy, including new contracts for GPs to work across larger populations and capitated year-of-care budgets [44]. Supporting evidence exists but is indirect: Bodenheimer and colleagues' study of high-performing US primary care practices identified team-based care, population management, and continuity as common features [45], and van Weel and Kidd argue that strengthening generalist-led primary care is essential to universal health coverage [46].

In our assessment, evidence on PCNs' clinical effectiveness remains early and mixed, and implementation has varied, with some networks achieving genuine multidisciplinary integration and others functioning mainly as administrative structures. Barriers to integration, including separate information systems, organisational cultures, contracts, and professional identities, should not be underestimated. Whole-system reforms of this kind are difficult to randomise, so stepped-wedge, natural-experiment, and interrupted time-series designs are more feasible ways to evaluate them.

The risk of poorly designed team expansion is fragmentation: the pharmacist manages the diabetes, the physiotherapist the osteoarthritis, the social prescriber the low mood, and nobody holds the whole picture. The GP's distinctive function is what might be called cognitive integration: the capacity to see across conditions, for example, to recognise that repeated consultations for vague physical symptoms may reflect domestic abuse. This capacity can be supported and measured. In the IRIS trial, practice-based training with a direct referral pathway increased identification and referral of women experiencing domestic violence [47]; referral rates to such pathways are one feasible indicator. The Declaration of Alma-Ata's vision of care brought as close as possible to where people live and work [48] implies proportionate funding for practices serving communities furthest from equitable access.

Continuity of care has unusually strong observational support. Pereira Gray and colleagues' systematic review identified 22 studies from nine countries; 18 (81.8%) reported significantly lower mortality with greater continuity [49]. Continuity can be measured routinely: the Usual Provider of Care (UPC) index, the most common measure in that review, and the St Leonard's Index of Continuity of Care (SLICC), which can be calculated from practice appointment data [50]. Any reformed model that sacrifices continuity for throughput would discard the best-evidenced mechanism of benefit primary care has.

Critical synthesis: common threads and research gaps

Four threads run across the six domains (Table 2). First, there is a consistent mismatch between what populations need and what the current model is designed to deliver; this reflects inherited structure, not failure by individual clinicians. Second, the proposed reforms are supported mainly by observational and policy evidence rather than prospective trials, partly because whole-system reform resists randomisation. Third, equity runs through every domain: those most affected by the model's shortcomings have the greatest clinical need, least digital access, and greatest language barriers. Fourth, international comparisons reinforce the English findings: stronger primary care is associated with better outcomes, and equitable access is both ethically necessary and plausibly cost-effective. That this evidence has existed for decades without producing proportionate structural change is itself a finding. 

**Table 2:** Summary of structural challenges, evidence, equity implications and proposed responses RCT: randomised controlled trial; PROMs: patient-reported outcome measures; FTE: full-time equivalent; GP: general practitioner; AI: artificial intelligence; ONS: Office for National Statistics; UK: United Kingdom; NHS: National Health Service; UPC: Usual Provider of Care; SLICC: St Leonard's Index of Continuity of Care.

Domain	Key evidence	Evidence strength	Equity implication	Proposed response	Measurable indicator
Multimorbidity	Barnett et al. [10]; Salisbury et al. (3D trial) [12]; Barker et al. [13]	Moderate: large cross-sectional, one RCT (null primary outcome), cohort	Earlier onset and more mental-physical comorbidity in deprived areas	Longer whole-person reviews; single care plan; outcome-based indicators replacing single-disease targets	Annual whole-person review completion; treatment burden PROMs; ambulatory care sensitive admissions
Workforce	Karuna et al. [15]; Verhoef and Blomme [16]; Long et al. [19]; Nussbaum et al. [20]	Moderate: systematic reviews of surveys and qualitative studies; longitudinal workforce data	1.4 fewer FTE GPs per 10,000 patients in most deprived areas	Retention-first strategy; protected time; support in high-deprivation practices	FTE GPs per 10,000 need-adjusted patients by deprivation decile; GP leaver rates
Technology and AI	Parker et al. [4]; Blease et al. [23]; Daneshjou et al. [26]; Obermeyer et al. [27]	Low to moderate: systematic review of observational studies; surveys; validation studies	Digital exclusion overlaps with clinical vulnerability; algorithmic bias	Guaranteed non-digital routes; equity audit before deployment	Consultation mode and access by age, deprivation, ethnicity and language; AI performance by subgroup
Migration and cultural competence	ONS [6,7]; Rechel et al. [30]; Doctors of the World [34]	Low for UK outcomes; moderate European evidence	Language, entitlement and registration barriers	Interpreter access standards; registration compliance; cultural safety for international recruits	Interpreter use per consultation needing one; wrongful registration refusals
Equity and funding	Hart [25]; Marmot et al. [36,37]; Starfield et al. [39]; Fisher et al. [41]	Moderate: cross-national observational and policy analyses	Funding and workforce do not track need	Reweight capitation for deprivation, multimorbidity and language need	Funding per need-adjusted patient by deprivation decile
Neighbourhood models and continuity	Fuller [42]; Department of Health and Social Care [44]; Pereira Gray et al. [49]	Strong observational for continuity; low for neighbourhood models at NHS scale	Fragmentation harms patients with complex needs most	Neighbourhood teams that protect named-GP continuity; prospective evaluation	UPC or SLICC continuity indices; stepped-wedge evaluation outcomes

What the literature does not yet provide is prospective evidence that specific reform models achieve their intended outcomes at NHS scale. Research priorities are: prospective evaluation of neighbourhood health models in diverse English settings using stepped-wedge or natural-experiment designs, with continuity, access by deprivation and language, and ambulatory care-sensitive admissions as outcomes; controlled evaluation of digital inclusion interventions; equity audits of AI tools in UK primary care; and studies of health outcomes in specific post-Brexit migrant populations in English primary care. Although the conclusions are framed for England, the principles may be relevant to other registered-list primary care systems.

This review has several limitations worth acknowledging. As a narrative rather than systematic review, literature selection was guided by thematic relevance rather than a pre-registered protocol, and screening was not performed independently in duplicate, introducing possible selection bias towards studies supporting our conclusions. No formal screening log was kept, and no formal risk-of-bias assessment was performed, although evidence strength is graded by design in Table 2. Some evidence is from Scotland, the wider UK, or other countries, which may limit transfer to England. Several proposed reforms rest mainly on observational, cross-national, and policy evidence, and their real-world effectiveness at NHS scale is unproven. Evidence on outcomes for specific post-Brexit migrant groups in UK primary care is sparse, which limits the strength of conclusions in that area and represents a research gap in its own right.

## Conclusions

The evidence reviewed suggests that general practice in England will require substantial structural reform to meet the demands of the next three decades. The current model is misaligned with the epidemiology of multimorbidity and ageing, has not adapted adequately to the post-Brexit change in the populations it serves, lacks safeguards to deploy digital innovation equitably, and rests on workforce and contractual foundations that incremental adjustment has not stabilised.

We propose four specific changes, each with a measurable indicator. First, contracts should reweight capitation for deprivation, multimorbidity, and language need, monitored by funding and FTE GPs per need-adjusted patient across deprivation deciles. Second, continuity should be protected within neighbourhood teams and reported routinely using the UPC index or SLICC. Third, interpreter access and digital inclusion should be resourced as core infrastructure, with consultation access monitored by age, deprivation, ethnicity, and preferred language, and every AI or digital access tool subject to equity impact assessment before and after deployment. Fourth, workforce policy should prioritise retention and cultural safety, including structured induction for international medical graduates, monitored by leaver rates and workforce distribution. The strongest evidence supports the second of these: continuity with a known doctor is consistently associated with lower mortality, and no reform should be designed in ways that undermine it.
